# Direct measurement of brake wear particles from a light-duty vehicle under real-world driving conditions

**DOI:** 10.1007/s11356-024-35879-y

**Published:** 2025-01-13

**Authors:** Tawfiq Al Wasif-Ruiz, Ricardo Suárez-Bertoa, José Alberto Sánchez-Martín, Carmen Cecilia Barrios-Sánchez

**Affiliations:** 1 Research Centre for Energy, Environment and Technology (CIEMAT), Avda. Complutense, 40, 28040 Madrid, Spain; 2 European Commission, Joint Research Centre (JRC), Via Enrico Fermi, 2749, 21027 Ispra, VA Italy

**Keywords:** Non-exhaust emissions, Brake wear particles, Nanoparticles, Direct measurement, Real-world driving conditions, Scanning electron microscope

## Abstract

As tailpipe emissions have decreased, there is a growing focus on the relative contribution of non-exhaust sources of vehicle emissions. Addressing these emissions is key to better evaluating and reducing vehicles’ impact on air quality and public health. Tailoring solutions for different non-exhaust sources, including brake emissions, is essential for achieving sustainable mobility. Studying emissions from vehicles in real-world scenarios provides a better understanding of their environmental impact compared to laboratory testing alone. This study presents findings on the direct measurement of brake particles and the characterization of this source of particulate matter in real-world conditions using a mobile laboratory. In situ measurements of particle concentration and size distribution showed good agreement with previous laboratory studies, indicating the suitability of the approach to investigate break particle emissions during real-world operation. The study demonstrates that particle size distributions can vary based on the temperature of the brake disk, which is influenced by the initial braking speed, with significant variations observed between speeds of 60, 80, 100, and 120 km/h. Particles with sizes between 6 and 523 nm were released into the air from the brake system, although it is likely that larger particles were also emitted but not captured due to the upper detection limit of the Engine Exhaust Particle Sizer. During harsh braking events, such as decelerations of 4.2 m/s^2^ from 120 km/h, a concentration of up 10^6^ (#/cm^3^) was measured for particles under 8 nm. Moreover, scanning electron microscope analysis revealed that nanoparticles are present in the form of agglomerates, whose shape can change depending on the formation process. Elements present in the particles comprised mainly iron, copper, and aluminium, indicating wear of the brake pad materials and disk components.

**Supplementary Information:**

The online version contains supplementary material available at 10.1007/s11356-024-35879-y.

## Introduction

Emissions of inhalable particulate matter (PM) from road transport are one of Europe’s most problematic pollutants due to their impact on human health. Fine particles, specifically particulate matter with an aerodynamic diameter of less than 2.5 µm (PM2.5), are particularly concerning because they can lead to cardiovascular and respiratory diseases due to their potential to cause lung damage (Amato et al. 2011; Dahl et al. 2006; Kwak et al. 2013; Timmers and Achten 2016). Given these significant health issues, there is increased interest in understanding the contribution of non-exhaust emissions from road transport, as these sources can further exacerbate the problem and are essential for a comprehensive approach to managing air quality.

Wu et al. (2021) indicate that non-exhaust emissions during tunnel measurements in the UK and China contribute 50–75% of PM10 and 15–40% of PM2.5, respectively. Additionally, they projected an increase in these emissions in the coming years if no control measures are implemented. Similarly, in their review, Grigoratos and Martini (2015) noted that non-exhaust can contribute over 50% of particulate matter in urban environments.

The main sources of non-exhaust emissions are brakes, tires, clutch, road surfaces, and the resuspension of road surface dusts (Beddows et al. 2016; Beddows and Harrison 2021; Liati et al. 2019; Mathissen et al. 2011; Timmers and Achten 2016). Non-exhaust’s particles are generated by various mechanical processes, such as abrasion or degradation of the materials (Gehrig et al. 2010; Grigoratos and Martini 2015; Mamakos et al. 2021; Niemann et al. 2020; Park et al. 2017). During a high temperature events, ultrafine particles are generated; thus, this indicates a second path of particle generation (Hesse et al. 2021; Mamakos et al. 2021; Mathissen et al. 2011; Niemann et al. 2020).

Brake wear PM is a complex mixture of particles with a very different size, which has received an increase attention over the last two decades. The contribution of brake wear particles in urban environments to non-exhaust PM10 could be around 55% by mass and up to 21% by mass to total traffic PM10 emissions (Grigoratos and Martini 2015; Niemann et al. 2020; Oroumiyeh and Zhu 2021). However, ultrafine, fine, and coarse particles could be originated by this non-exhaust source (Bondorf et al. 2023; Mamakos et al. 2021; Niemann et al. 2020), showing that particles from brakes are emitted in different size modes (Bondorf et al. 2023).

Globally, non-exhaust traffic emissions, including brake wear, have been recognized as a significant contributor to ambient particulate matter pollution. Piscitello et al. (2021) reported that non-exhaust traffic emissions could represent up to 90% of total traffic-related particulate matter, emphasizing the need for standardization and mitigation efforts. This broad category includes emissions from brakes, tires, road surfaces, and the resuspension of road dust. Focusing specifically on brake wear, Grigoratos and Martini (2015) noted that brake wear could contribute up to 55% of total particulate matter emissions in urban environments. More studies have reinforced these findings; for instance, Harrison et al. (2012) found that brake wear particles account for approximately 48 to 62% of total particulate matter emissions, depending on traffic conditions. Collectively, these finding emphasize the critical need for targeted interventions to mitigate the environmental impact of brake wear particles.

Kukutschová et al. (2011) studied the wear particles distribution released from low-metallic automotive brakes using a brake dynamometer simulation. They found in 2011 that the concentration of ultrafine particles increases when the temperature of the rotor is about 300 °C. In contrast, Oroumiyeh and Zhu (2021), who studied the effect of vehicle’s mass and braking intensity on brake particle based on on-road data collection, found a unimodal mass size distribution with a mode diameter of 3–4 µm.

Finally, Niemann et al. (2020) used an enclosed brake dynamometer in a laboratory setting to study the influence of disk temperature on different particle size emissions and found emissions of ultrafine, fine, and coarse particles. Based on their results, the size distribution and particle emissions of ultrafine particles appear to be temperature-dependent. However, these particles decrease at a certain temperature, indicating a thermal aging effect on the pad material due to decomposition. For fine and coarse particles, temperature is not a major influencing factor. This study suggests that decomposition primarily affects the behaviour of fine and coarse particles.

There is still a gap in the results regarding brake particle emissions (Oroumiyeh and Zhu 2021; Park et al. 2018). Therefore, it is necessary to characterize the types and sizes of brake particles, as well as analyse wear debris and their environmental impact (Dahl et al. 2006; Kukutschová et al. 2011). The first step in categorizing and analysing break particles is particle measurement. The lack of factual data in some studies is due to the absence of a method to measure the contribution of break wear PM on the environment (Grigoratos et al. 2020; Sachse et al. 2014).

The current work aims to go beyond the study of the single component (the break) in the laboratory and investigates the possibility to reliably measure the emissions of brake particles from vehicles during real-world operation using an on-board laboratory for this purpose. This research sets itself apart from previous studies by concentrating on real-world driving scenarios, where key variables like fluctuating speeds, brake temperatures, and diverse road surfaces come into play.

Laboratory conditions often fail to capture the complexity of real-world driving, where factors such as sudden braking, varying speeds, and different road textures can significantly influence brake wear and particle emissions. For instance, higher vehicle speeds can lead to increased brake temperatures, which in turn can cause greater thermal decomposition of brake materials and higher emissions of ultrafine particles. Similarly, the interaction between the brake system and different road surfaces can affect the generation and dispersion of particles.

A significant contribution of this work is the use of a polycarbonate isolator that covers the rim, minimizing environmental interferences and ensuring that the measured particles originate directly from the brake system. This innovative method of on-road sampling and measurement provides data that are more representative of actual vehicle operating conditions, something not achieved with traditional laboratory studies. It also assesses online the concentration and size distribution of the particles emitted by the break under different conditions and offline their morphological and chemical characteristics.

## Materials and methods

Break particles from a Skoda Yeti were measured and sampled during a series of on-road tests under a set of different real-world braking conditions. The car was chosen for the tests as an example of a passenger vehicle that could be used in urban, rural, and motorway areas. Its laden mass was 1340 kg, which increased to 1885 when the measurement equipment was included. The vehicle’s brake system was the original equipment manufacturer (OEM). However, the exact composition of the brake pads is not available due to proprietary restrictions that limit access to this type of information. To address this, we conducted reference measurements directly on both the brake pads and the brake disk to establish baseline data on emissions. The fundamental compounds identified in the brake pads and disk components include iron, copper, and aluminium, which are indicative of the materials used in these components.

The on-road tests followed an in-house cycle (called hereinafter “hot braking”) that simulated harsh braking resulting from a spontaneous brake application (bringing the vehicle to a standstill in 8 s, corresponding to a deceleration of 4.2 m/s^2^ from 120 to 0 km/h, and 2.1 m/s^2^ from 60 to 0 km/h), due to traffic on the highway or during rural driving. The “hot braking” cycle was chosen due to its ability to simulate harsh braking conditions, allowing for the evaluation of brake system performance and associated emissions in critical situations. This choice is crucial because harsh braking can significantly impact the generation of brake wear particles.

The test is termed “hot” because, prior to the actual braking events, the brake system is preheated to over 250 °C through 15 consecutive braking events at different speeds. This preheating replicate demanding braking scenarios, such as continuous braking on steep downhill roads, heavy stop-and-go traffic, or emergency stops at high speeds, where brake temperatures can rise significantly. These conditions were chosen to simulate situations where, after harsh braking, vehicles may enter urban areas with the brake system still hot. Under these circumstances, even light braking can produce significant particle emissions, which directly affect air quality and public health, especially in densely populated areas. This approach allows for an evaluation of brake system performance and associated emissions in critical scenarios, providing data that reflects real-world driving conditions.

Compared to the WLTP-Brake cycle, which is designed to represent typical urban, rural, and motorway driving conditions with a focus on average driving patterns, the “hot braking” cycle specifically targets intense braking scenarios. The WLTP-Brake cycle includes a variety of braking events at different speeds and decelerations, but it does not necessarily replicate the high-temperature conditions that can occur during prolonged or severe braking. The “hot braking” cycle was chosen to ensure that the brake system is tested under the most demanding conditions, providing insights into the maximum potential emissions of brake wear particles. This approach complements the WLTP-Brake cycle by focusing on the upper limits of brake system performance and emissions, which are critical for understanding the full range of real-world driving conditions.

Prior to each test, background particle levels were measured with the car stationary to ensure a clear distinction between ambient air particles and those generated by the braking events. These background levels were recorded for 5 min before each test. The speed sequence followed during the preheating process was as follows: three braking events from 60 to 0 km/h in 8 s, three from 80 to 0 km/h in 8 s, three from 100 to 0 km/h in 8 s, and finally, three from 120 to 0 km/h in 8 s. Once the brake system was heated, the measurements under the “hot braking” cycle began. This cycle involved repeating the same conditions used in the preheating process, measuring the particles emitted during those braking events and reaching a maximum disk temperature of 380 °C.

The brake particles from the vehicle’s front left axle were sampled through an INOX + Tygon® tube attached to the backing plate, near the callipers and brake pads, specifically at the lower lateral side of the brake pad. The tube was positioned to capture particles emitted directly from the braking interface. The sampling was performed using an Engine Exhaust Particle Sizer (EEPS™) 3090, which measures the concentration and particle size distribution in the range from 5.6 to 560 nm. The EEPS operates on the principle of electrical mobility, where particles are charged and then passed through an electric field. Based on their size, particles acquire different electrical charges and experience different forces in the field, allowing them to be classified by size. Using this information, the dynamic behaviour of the particles was analysed in the different situations resulting from the cycle.

The sampling position was selected to provide a direct path from the brake to the sampling tube, minimizing particle loss. This study focused specifically on ultrafine particle emissions, and the current sampling design does not target fine-sized brake wear particles (PM10 and PM2.5).

One of the key innovations in this study is the use of a polycarbonate isolator that covers the rim to minimize potential environmental interference, ensuring that the particles measured come directly from the brake system. Additionally, the brake system’s inherent design provides a degree of shielding on the inner side of the rim and brake assembly, limiting the ingress of external particles. This is achieved through the close fit of the brake components and the positioning of the brake pads and callipers, which act as a barrier. Furthermore, the dynamic movement of the brake components during operation further reduces the likelihood of external contamination affecting the measurement of brake wear particles.

To verify this shielding effect, we conducted additional trials where the vehicle was driven at a constant speed, and the brake was applied using only the handbrake. Notably, the concentrations measured during these tests were comparable to typical ambient particle levels in the area where the trials are conducted (total particle concentration of 2.0 × 10^5^ #/cm^3^), suggesting that the inner area is effectively shielded and that no significant interference occurred from this section.

In addition to measuring the particles online in the real world, seven filters were used for offline measurements. The filters were collected during dedicated tests performed using only braking events from 120 to 0 km/h. This allowed the collection of particles at high temperatures (see the “Morphology and chemical composition” section). The filters, consisting of a carbon filter supported by a 150-mesh copper grid, were placed close to the brake pads to maximize the collection of particles from that area. With these filters, the morphology of the particles was studied offline using a scanning electron microscope (SEM) and an energy-dispersive X-ray spectroscopy (EDS).

SEM analysis provides high-resolution images of the surface morphology and structure of the brake particles, allowing for a detailed examination of their shapes, sizes, and distribution. EDS, on the other hand, offers compositional analysis by detecting the elemental makeup of the particles. This combination is particularly valuable because it allows us to correlate the physical characteristics of the particles with their chemical composition, which is essential for understanding the wear mechanisms and sources of the particles.

The EEPS was not used during these tests to avoid the potential cooling effect on the brake system during the placement of the filters, which could alter the particle generation process at high temperatures. By using SEM and EDS, the study could characterize particle morphology and composition in a controlled, offline setting, complementing the dynamic, online measurements.

Additionally, to gain more insights into the brake system’s performance and the effect of the break temperature on the emissions and physicochemical properties of the particles, temperature profiles were measured at various critical locations. To this end, type K thermocouples, with an accuracy of ± 1 °C within the range of − 200 to 1372 °C, were used. These thermocouples were integrated into the brake pads, brake calliper, and brake disk. They recorded temperature data at a rate of twice per second, ensuring precise monitoring of the thermal behaviour of the brake components during operation.

The K-type thermocouple specifically designed for brake disks was mounted onto the disk using a custom-designed set of plates that were attached to the vehicle’s control arm (suspension arm). This setup allowed the thermocouple to remain in firm contact with the disk’s surface, ensuring temperature measurements during rotation while minimizing any interference with the braking performance. The thermocouple was positioned at the centre of the disk to capture representative temperature data. Although the protocol outlined in UN GTR24 was not specifically followed during the installation process, the methods used ensured a stable attachment of the thermocouples and temperature readings. The setup closely resembles the standardized methods, utilizing a contact sensor type K that was securely fixed, maintaining its position and ensuring continuous contact with the disk surface throughout the tests.

Levene’s test was used to assess the equality of variances across the different groups by looking into deviations from normality, which was considered appropriate for our set of data. The null hypothesis for Levene’s test posits that the population variances are equal across all groups, whereas the alternative hypothesis suggests that at least one group has a different variance. Additionally, we employed ANOVA (analysis of variance) to compare the population means across these groups. The method tests differences in means when dealing with more than two groups. In this case, the null hypothesis for ANOVA asserts that all group means are equal, while the alternative hypothesis indicates that at least one group mean differs.

## Results and discussion

### Size distributions

**Fig. 1:**
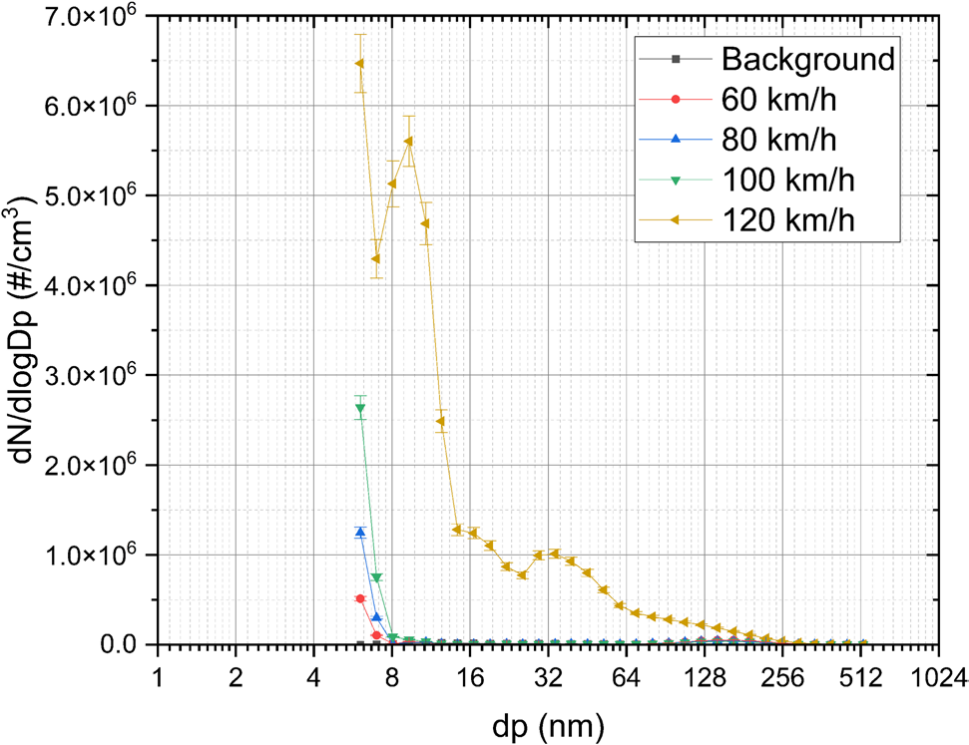
Average concentrations at different size distributions. Tests: 60, 80, 100, and 120 km/h

The average concentrations of particles for each diameter measured in the braking system during the “hot braking” cycle are shown in Fig. 1. At a significance level of 0.05, both the population variances (tested using Levene’s test) and the population means (tested using ANOVA) show statistically significant differences.

A concentration of 10^6^ (#/cm^3^) was reached by the EEPS at all the investigated speeds (except 60 km/h) for particles with a diameter of 6 nm. This is in agreement with Bondorf et al. (2023), who examined brake particle emissions from a test cycle on a chassis dynamometer using an EEPS and observed that 10^6^ (#/cm^3^) particles were emitted as ultrafine particles “with a diameter of approximately 10 nm” produced by thermal processes. For particles with a diameter of 8 nm, the concentration drops to levels close to the background for all the speeds investigated, except at 120 km/h. At this speed, although the concentrations decreased with the particle size, for which concentrations, although decreased with size of the particles, remained above 10^6^ #/cm^3^ for most sizes until approx. 50 nm.

The second peak observed at 120 km/h could be attributed to the higher initial kinetic energy at this speed (Grigoratos and Martini 2015), leading to more severe braking and, consequently, higher brake pad temperatures. These elevated temperatures, in turn, result in greater thermal decomposition and wear, generating a larger quantity of ultrafine particles. This aligns with laboratory-based studies, such as those by Bondorf et al. (2023), where the majority of brake wear particles were found in the ultrafine range, and predominantly generated by thermal processes.

However, this second peak observed in our real-world study at 120 km/h underscores the importance of investigating real-world driving scenarios. In these settings, higher speeds seem to exacerbate particle emissions beyond what is commonly observed in controlled laboratory environments, likely due to the complex and dynamic nature of on-road braking.

As can be seen, particle concentration increases with higher initial braking speeds, with 120 km/h resulting in significantly higher concentrations than other breaking speeds, regardless of particle size. The highest concentration of ultrafine brake particles was recorded at 6 nm during the 120 km/h tests, while the lowest concentration was recorded at 60 km/h.

**Fig. 2:**
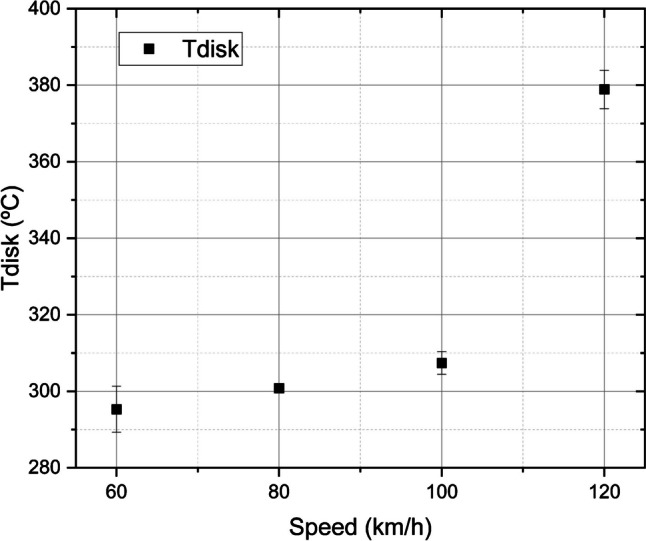
Final temperature of the brake disk at the end of braking. Tests: 60 km/h ± 295 °C, 80 km/h ± 301 °C, 100 km/h ± 307 °C, and 120 km/h ± 379 °C

Figure 2 illustrates how the final disk temperature (Tdisk) rises with vehicle speed at the end of braking. It shows that the temperature of the brake disk increases as with the vehicle deceleration (i.e., higher temperatures at 120 km/h and lower temperatures at 60 km/h). As the break disk’s temperature rises, the generation of finer particles also increases. These observations are in line with Mathissen et al. (2011), who reported a significant increase in particle emissions with increased deceleration due to the elevated temperatures achieved in the brake system during evaporation and decomposition processes.

Hence, it should be noted that although the results are presented for the different speeds studied, as this is the parameter that could be varied during testing, the differences observed were linked to the differences in temperatures resulting from these braking events.

As shown in Fig. 2, the final Tdisk drastically changed in slope at a speed of 120 km/h. The behaviour of Tdisk (see Figure S1 in the Supplementary Information) at a speed of 120 km/h ± 379 °C explains why there is a sudden, high increase in particle emissions when the size is 6 nm. At this temperature, the average concentration of 6 nm particles increases less gradually, leading to a jump in the concentration of particles of this size. This finding is consistent with previous studies by Hesse et al. (2021), who observed in an inertia dynamometer a critical temperature threshold in disk temperature, causing a “hot band” and the formation of nanoparticles as a result. Similarly, Niemann et al. (2020) studied the influence of disk temperature on ultrafine particle emissions from passenger cars using an enclosed brake dynamometer. Their findings indicate that the size distribution of the particles presents a temperature dependence.

In addition, the elevated temperatures observed suggest that various thermal decomposition processes are occurring within the brake pad material. As the temperature surpasses 250 °C, the friction materials begin to degrade, triggering chemical alterations within the organic components of the brake pads. This includes the breakdown of organic binders and the subsequent release of volatile compounds (Elzayady and Elsoeudy 2021; Mathissen et al. 2011), both of which contribute significantly to the formation of ultrafine particles. Moreover, while these thermal processes are well-documented in controlled laboratory conditions, it is essential to emphasize that they are also closely tied to real-world driving scenarios, where harsh braking at high speeds can result in elevated particle emissions.

Finally, the impact of these high temperatures on both brake wear efficiency and the overall longevity of the braking system should not be underestimated. The high brake disk temperatures recorded may lead to glazing of the brake pads. This glazing phenomenon reduces friction efficiency, which means that more force is required to achieve the same braking effect, leading to accelerated wear. Consequently, this can shorten the lifespan of both the brake pads and disks, especially when subjected to frequent high-temperature braking events (Elzayady and Elsoeudy 2021). Additionally, the increased wear can result in more frequent maintenance and replacement of brake components, impacting the overall cost and reliability of the braking system.

**Fig. 3:**
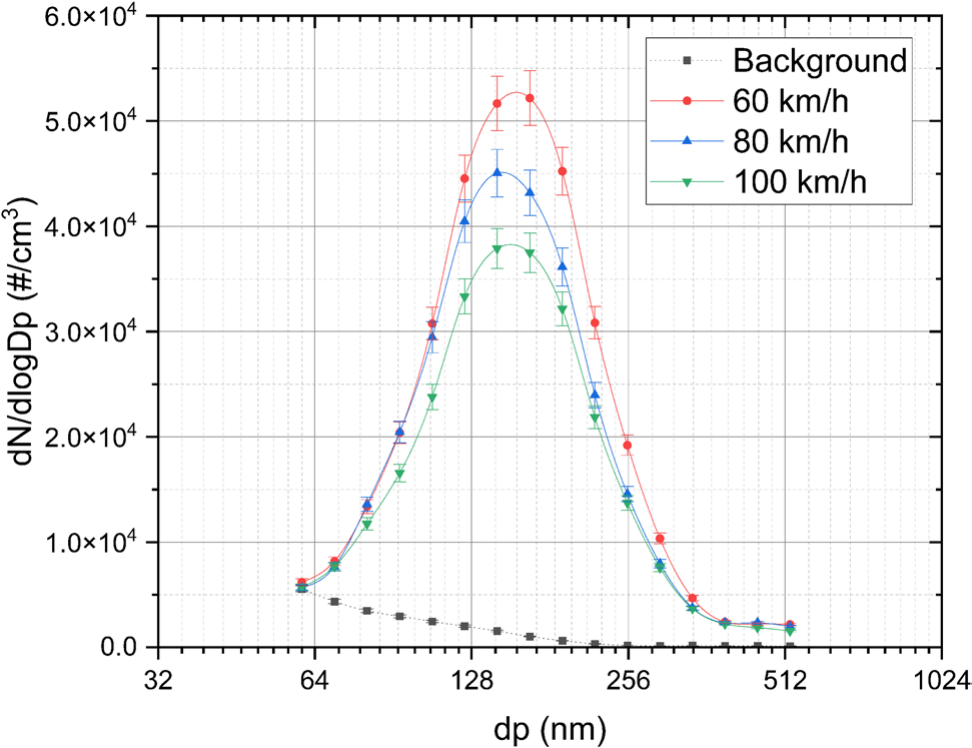
Average concentrations at different size distributions. Tests: 60 km/h ± 295 °C, 80 km/h ± 301 °C, and 100 km/h ± 307 °C

For a clearer understanding of the size distribution of brake particles during the “hot braking” cycle, Fig. 3 illustrates the size distribution excluding the initial speed of 120 km/h ± 379 °C and includes data for larger particle diameters.

Figure 3 illustrates the size distribution of particles measured at three different disk temperatures. A prominent single mode around 150 nm in diameter is observed at 60 km/h ± 295 °C, 80 km/h ± 301 °C, and 100 km/h ± 307 °C, exceeding the background size distribution, which is normally around 1.0 × 10^3^ #/cm^3^ at that size. This mode decreases in concentration as the initial braking speed increases, coinciding with an increase in peak concentration at 6 nm particle size. This mode is absent at 120 km/h ± 379 °C. Considering that, as shown in Fig. 1, at this speed, there are multiple modes at varying diameters. This indicates that the temperature of the disk affects the size distribution curve of brake particles.

This is in agreement with Garg et al. (2000), who analysed brake particulate emissions from various brake pads on a dynamometer using a micro-orifice uniform deposit impactor (MOUDI) and concluded that the particle size distribution varies with temperature. Their study suggests that the increase in temperature causes a decrease in the large particle fraction. The elevated temperatures are sufficient to degrade brake materials, volatilizing them from the brake pads and contributing to the formation of smaller particulates.

Furthermore, Beji et al. (2020), who studied non-exhaust particle emissions under various driving conditions on a chassis dynamometer, identified a bimodal distribution in brake emissions with an accumulation peak centred around 200 nm. Additionally, Mathissen et al. (2011) observed a unimodal size distribution with a diameter of approximately 11 nm. Similarly, Kwak et al. (2014), who examined braking events under deceleration conditions both on a proving ground and using a road simulator, reported a mode at 10 nm.

As shown above, the results obtained from particulate emissions measured from the brake — specifically in terms of concentration, correlation with disk temperature, deceleration, speeds, and size distribution — during on-road tests under real-driving conditions were in good agreement with previous studies performed in laboratory settings using isolated brake systems and/or under highly controlled conditions. This suggests that the developed setup provides reliable sampling and measurement of brake emissions, effectively bridging the gap between controlled laboratory experiments and real-world operations.

### Morphology and chemical composition

**Fig. 4:**
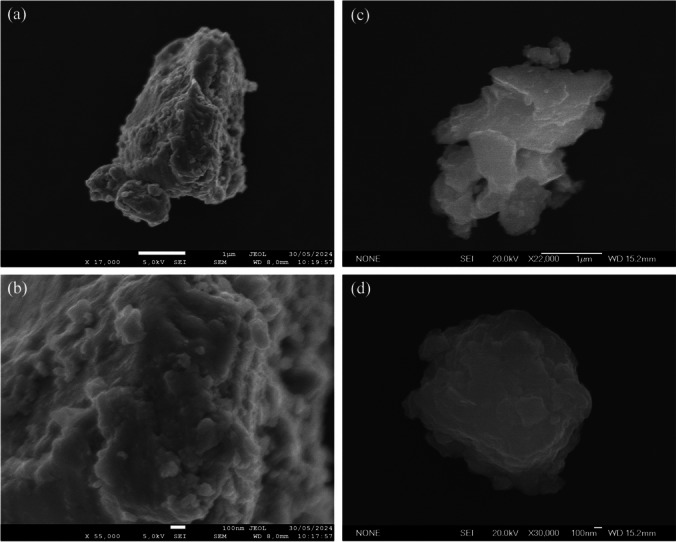
SEM images of particles under the two Tdisk conditions examined. Images **a** and **b** correspond to a maximum Tdisk of 257 °C. Images **c** and **d** correspond to a maximum Tdisk of 307 °C

The morphology and chemical composition of brake particles were analysed using SEM and EDS. Figure 4 displays particles captured during braking events from 120 to 0 km/h. Four of the filters reached a maximum Tdisk (see Figure S2 in the Supplementary Information) of 257 °C, while the other three reached a maximum Tdisk (see Figure S2 in the Supplementary Information) of 307 °C, enabling the study of the temperature’s effect on particle morphology.

Based on SEM images, the morphology of brake particles differs between the two disk temperatures. In both cases, particles occur in the form of aggregates, and single particles were not found at this size. However, the particles from the test at Tdisk of 257 °C, images (a) and (b) from Fig. 4, show more aggregates with irregular and rough shapes compared to those obtained during the test at a maximum Tdisk of 307 °C. This difference is likely due to the lower disk temperature and the dominance of mechanical formation processes (Woo et al. 2023). Specifically, at the lower disk temperature, mechanical wear predominantly drives particle formation since the friction materials do not reach sufficiently high temperatures to undergo thermal softening or melting. As a result, the materials break off as larger, jagged fragments, which, in turn, lead to particles with rougher and more irregular shapes. Furthermore, these particles are more prone to aggregation because of their rough surfaces and the absence of thermal processes that would otherwise produce smoother, more uniform particles. On the other hand, at the higher disk temperature of 307 °C, thermal degradation becomes more significant. The heat softens or melts the friction materials, thereby leading to the formation of smaller, smoother, and more spherical particles. Consequently, due to their smoother surfaces, these particles have a reduced tendency to aggregate, resulting in more dispersed individual particles.

This is in agreement with Liati et al. (2019), who analysed morphological and chemical properties of airborne particulate matter emissions from vehicle brakes using a brake test bench and electron microscopy at disk temperatures between 150 and 300 °C. The study indicates that particles typically occur in the form of aggregates across different size ranges (from coarse to ultrafine particles). The fine particles found (dp ~ 0.141–2.056 µm) were usually equidimensional with irregular shapes, and single particles occurred very seldom.

However, image (d) from Fig. 4 shows a different morphology. This aggregate, likely generated due to the high Tdisk, had a round shape. This behaviour was reported by Woo et al. (2023), who characterized brake particles from two different brake pads under normal and harsh braking conditions using a brake dynamometer. Their study found that under harsh braking conditions, nanoparticles with a diameter of around 50 nm exhibited a round morphology, which they attributed to the volatilization and nucleation of the organic compound in the pads. These nanoparticles then adhered to larger particles at elevated pad temperatures. Although Woo et al. (2023) focused on pad temperatures, the same effect can be inferred in the present work due to the correlation between higher disk temperatures and increased pad temperatures, both of which contribute to the observed morphological changes.

This distinction between particle sizes and shapes at varying temperatures carries broader implications for both environmental and health risks. On the one hand, the larger, irregular particles formed at lower temperatures tend to settle in the upper respiratory tract, where they can contribute to respiratory irritation and discomfort (Kelly and Fussell 2012; Oberdörster et al. 2005). On the other hand, the smaller, spherical particles generated at higher temperatures pose a more severe risk. Due to their ultrafine nature, they are more likely to bypass the body’s natural defences and penetrate deep into the lungs, potentially reaching the bloodstream. Once in the circulatory system, they can trigger a range of systemic health effects (Kelly and Fussell 2012; Oberdörster et al. 2005).

To fully characterize brake wear particles, elemental analysis was performed on samples collected at 257 °C and 307 °C. The most abundant metallic element in all samples was iron (Fe), followed by copper (Cu), aluminium (Al), and zinc (Zn). Iron, copper, and zinc have been reported to be present in high concentrations in brake pads (Kukutschová et al. 2011; Liati et al. 2019; Wahlstro 2010). The presence of iron in the particles was most likely related to the brake disk. On the other hand, copper and aluminium commonly originate from the brake pads (Kukutschová et al. 2011; Liati et al. 2019; Wahlstro 2010).

Other elements found included magnesium (Mg), silicon (Si), sulphur (S), chromium (Cr), tin (Sn), barium (Ba), and zirconium (Zr). These elements were found in low quantities (< 1 wt. %), and Zr was found only in particles collected at a Tdisk of 257 °C. This particle chemistry is in agreement with Liati et al. (2019), who found these amounts of elements in two different types of disks and commercially available brake pads at different particle sizes on a brake test bench at disk temperatures between 150 and 300 °C.

The presence of these metallic elements, particularly at elevated temperatures, introduces additional environmental risks. Metals such as copper and zinc, for example, are known to be highly toxic to aquatic ecosystems once they are deposited into water bodies through atmospheric deposition (Fu et al. 2016). Therefore, the increase in finer particles observed at higher disk temperatures further intensifies this threat.

The results obtained from the morphology study and elemental analysis together with the good agreement with previous studies performed in the laboratory when studying particles originating from disks and brake pads suggest that the setup developed to collect particles on filters during on-road testing allows to collection of break particles in isolation from other potential non-exhaust sources. This will allow the investigation of the health and environmental risks associated with brake wear particles under wider and more realistic conditions, making it possible to assess their true impact during real-world operation.

## Conclusions

In this study, we introduce a novel method for sampling and measuring brake wear particles on-road under various real-world driving conditions.

Our findings indicate that brakes generate a large amount of ultrafine particles, reaching a concentration of 10^6^ #/cm^3^ for particles with a diameter of 6 nm at speeds above 60 km/h. Moreover, the results showed that concentration size distributions exhibit different modes that depend on the temperature of the disk, which varied with the initial speed of the studied breaking events. Additionally, as the temperature of the disk increases, the concentration of ultrafine particles correspondingly increases. Therefore, the temperature of the disk, which is influenced by the initial braking speed, plays a crucial role in determining the particle size distribution.

The results of the SEM and EDS suggest that particles occur in the form of aggregates. Depending on the formation — whether mechanical and/or thermal — the shape of the particles can vary from irregular and rough to round. Regarding the composition of the particles, the most abundant metallic element is iron, which primarily originates from the brake disk. This is followed by copper and aluminium, which originate from the brake pads.

The good agreement obtained between results and reported for laboratory methodologies under controlled conditions with single elements such as the brake suggest that the system used is capable of isolating break particles allowing measurement of particles emissions on-line in real-world operation and to effectively characterize off-line particle morphology and composition using scanning electron microscopy with elemental analysis system. Understanding and addressing these emissions in the real-world is essential to for a more precise evaluation of the overall impact of vehicles on air quality and public health. By focusing on non-exhaust emissions, key strategies to minimize pollution exposure and mitigate related health risks can be further developed.

The practical implications of this work are significant, as they open the possibility of developing emission factors for brake wear particles, which could be expressed in terms of mass or number per kilometre travelled. These factors would represent valuable information for regulatory agencies and vehicle manufacturers, as they would enable the implementation of effective measures aimed at reducing particle emissions from vehicles. Furthermore, future research could involve applying these findings to a broader range of vehicles and driving conditions, as well as integrating this data into models that predict the impact of brake wear on urban air quality. This work could also complement the WLTP-Brake cycle by providing additional real-world data to enhance the comprehensiveness of emission assessments. Specifically, considering scenarios where vehicles transition from high-speed driving to 0 km/h or downhill driving to urban areas with hot brake systems could help address the increased emissions observed under these conditions.

## Supplementary Information

Below is the link to the electronic supplementary material.

Supplementary file1 (DOCX 957 KB)

## Data Availability

All authors confirm that the data, materials, and software code used in this study are available upon request and comply with relevant field standards and journal policies. Additionally, supplementary information, including figures that may provide valuable insights into the article, is included in the Supplementary Information file.
