# Correction to: Direct measurement of brake wear particles from a light‑duty vehicle under real‑world driving conditions

**DOI:** 10.1007/s11356-025-37053-4

**Published:** 2025-10-07

**Authors:** Tawfiq Al Wasif‑Ruiz, Ricardo Suárez‑Bertoa, José Alberto Sánchez‑Martín, Carmen Cecilia Barrios‑Sánchez

**Affiliations:** 1 Research Centre for Energy, Environment and Technology (CIEMAT), Avda. Complutense, 40, 28040 Madrid, Spain; 2 European Commission, Joint Research Centre (JRC), Via Enrico Fermi, 2749, 21027 Ispra, VA Italy; 3 Universidad Politécnica de Madrid (UPM), C/José Gutiérrez Abascal 2, 28006 Madrid, Spain

**Correction to: Environmental Science and Pollution Research (2025) 32:2551–2560**

10.1007/s11356-024-35879-y

Tawfiq Al Wasif‑Ruiz has a 2nd affiliation: Universidad Politécnica de Madrid (UPM), C/ José Gutiérrez Abascal 2, 28006 Madrid, Spain.

