# Supplementary material for: Direct measurement of brake wear particles from a light-duty vehicle under real-world driving conditions
Source: Environ Sci Pollut Res Int. 2025 Jan 13;32(5):2551–60. doi: 10.1007/s11356-024-35879-y (PMC11802706; doi:10.1007/s11356-024-35879-y)
Supplement: Supplementary file 1 — Supplementary file1 (DOCX 957 KB) [file 11356_2024_35879_MOESM1_ESM.docx]

**Direct measurement of brake wear particles from light-duty vehicle under real-world driving conditions**

**Environmental Science and Pollution Research**

**Tawfiq Al Wasif-Ruiz^1*^; Ricardo Suárez-Bertoa^2^; José Alberto Sánchez-Martín^1^; Carmen Cecilia Barrios-Sánchez^1^**

^1^ Research Centre for Energy, Environment and Technology (CIEMAT), Avda. Complutense, 40, 28040 Madrid, Spain

^2^ European Commission, Joint Research Centre (JRC), 21027 Ispra, Italy

*Corresponding author: tawfiq.alwasif@ciemat.es

Figure S1. Temperature profiles for the four tested speeds, capturing the three braking events conducted at each speed.





Figure S2. Temperature profile for the tests at 120 to 0 km/h performed with filters used for morphological and chemical analysis.
